# Complete chloroplast genome of *Macadamia integrifolia *confirms the position of the Gondwanan early-diverging eudicot family Proteaceae

**DOI:** 10.1186/1471-2164-15-S9-S13

**Published:** 2014-12-08

**Authors:** Catherine J Nock, Abdul Baten, Graham J King

**Affiliations:** 1 Southern Cross Plant Science, Southern Cross University, Military Road, NSW, Lismore, 2480, Australia

## Abstract

**Background:**

Sequence data from the chloroplast genome have played a central role in elucidating the evolutionary history of flowering plants, *Angiosperma*e. In the past decade, the number of complete chloroplast genomes has burgeoned, leading to well-supported angiosperm phylogenies. However, some relationships, particulary among early-diverging lineages, remain unresolved. The diverse Southern Hemisphere plant family Proteaceae arose on the ancient supercontinent Gondwana early in angiosperm history and is a model group for adaptive radiation in response to changing climatic conditions. Genomic resources for the family are limited, and until now it is one of the few early-diverging 'basal eudicot' lineages not represented in chloroplast phylogenomic analyses.

**Results:**

The chloroplast genome of the Australian nut crop tree *Macadamia integrifolia *was assembled *de novo *from Illumina paired-end sequence reads. Three contigs, corresponding to a collapsed inverted repeat, a large and a small single copy region were identified, and used for genome reconstruction. The complete genome is 159,714bp in length and was assembled at deep coverage (3.29 million reads; ~2000 x). Phylogenetic analyses based on 83-gene and inverted repeat region alignments, the largest sequence-rich datasets to include the basal eudicot family Proteaceae, provide strong support for a Proteales clade that includes *Macadamia, Platanus *and *Nelumbo*. Genome structure and content followed the ancestral angiosperm pattern and were highly conserved in the Proteales, whilst size differences were largely explained by the relative contraction of the single copy regions and expansion of the inverted repeats in *Macadamia*.

**Conclusions:**

The *Macadamia *chloroplast genome presented here is the first in the Proteaceae, and confirms the placement of this family with the morphologically divergent Plantanaceae (plane tree family) and Nelumbonaceae (sacred lotus family) in the basal eudicot order Proteales. It provides a high-quality reference genome for future evolutionary studies and will be of benefit for taxon-rich phylogenomic analyses aimed at resolving relationships among early-diverging angiosperms, and more broadly across the plant tree of life.

## Background

Chloroplasts are the plastid organelles responsible for photosynthesis, and their genomes have proven to be a valuable resource for plant phylogenetics, population genetics, species identification and genetic engineering. High-throughput next generation sequencing (NGS) technologies have led to a rapid growth in the number of available chloroplast (cp) genomes, including representatives of most major lineages of green plants, *Viridiplantae *[1]. The quadripartite structure of the plant cp genome is highly conserved, with an inverted repeat region separating the small and large single repeat regions in most species [2].

Molecular phylogenomic studies utilising cp genome sequence data from the genes and slowly-evolving inverted repeat regions have been applied to unravel the deep-level evolutionary relationships of plant taxa [1-5], producing robust phylogenies that are corroborated by sequence data from mitochondrial and nuclear genomes [6]. Although cp genome phylogenies have been enormously important in resolving relationships among the flowering plants *Angiospermae*, the position of some lineages remains unresolved. Relationships among early-diverging lineages, including basal angiosperms, *Magnoliidae *(magnoliids), *Monocotyledoneae *(monocots) and basal *Eudicotyledoneae *(eudicots) have been among the most problematic due to rapid diversification early in the history of flowering plants [7]. Increased taxon sampling, particularly for taxa representing deep-level divergences, may provide resolution. [8,9].

The basal eudicot order Proteales contains the families Nelumbonaceae, Platanaceae and Proteaceae [10]. Fossil evidence and fossil-calibrated molecular dating indicate family-level divergence within the order by the early Cretaceous, over 110 million years ago [11]. There is evidence for long-term morphological and molecular stasis in the Nelumbonaceae and Platanaceae, and the only extant genera *Nelumbo *and *Platanus *are both regarded as 'living fossils' [12]. By contrast, the Southern Hemisphere family Proteaceae is morphologically and ecologically diverse. Approximately 79 genera and 1700 species are recognised, including the Australian *Banksia *and *Macadamia *and African *Protea*. Current distribution is the result of both vicariance during Gondwanan breakup and long-distance dispersal [13].

The Proteaceae exhibits remarkably variable levels of endemism and species-richness, notably in the Mediterranean climate biodiversity hotspots of Southwest Australia and the Cape Floristic Region [14,15]. It is, therefore, a family of great interest for studies of speciation, diversification, biogeography and evolution [16-18]. However, genomic resources for the Proteaceae are limited and little is known of the composition and organisation of the genomes and their evolution. Here, as part of an ongoing effort to establish a comprehensive understanding of the macadamia genomes, we present the complete and annotated DNA sequence for the chloroplast from *Macadamia integrifolia*, to our knowledge the first in the Proteaceae. Given that the closest reference sequences of *Platanus *and *Nelumbo *are over 100 million years divergent, the *Macadamia *cp genome was assembled *de novo *at deep coverage.

## Results

### *De novo *genome assembly

After trimming for low quality bases and adapter sequences, there were 1.54 × 10^8 ^reads with an average read length of 105 base pairs (bp). *De novo *assembly produced 540,582 contiguous sequences (contigs) with an N50 of 2,540. The maximum and average contig lengths were 300,523 and 1,032 respectively. Three chloroplast contigs were identified, with greatest similarity to *Platanus occidentalis *based on total alignment score and percentage sequence identity. These contigs totalled 133,617 bp in length and corresponded to the large single copy (88,300 bp), small single copy (18,888 bp) and a double-coverage, collapsed consensus of the inverted repeat regions (26,429). They were aligned to the *Platanus *cp genome using MUMmer as a starting point to order and assemble the draft genome (Fig. S1 in Additional File 1). The single collapsed inverted repeat (IR) contig was separated into two repeat regions. Assembly of the two IR and the large single copy (LSC) and small single copy (SSC) contigs covered the complete sequence without gaps. Iterations of assembly, realignment and editing using BWA, MUMmer and Gap5 were performed to complete the genome assembly. Sanger sequences spanning the inverted repeat and *de novo *contig junctions confirmed those in the final assembly. Reference mapping of paired-end reads was used to determine quality and coverage of the finished *Macadamia *cp genome. Following re-assembly of reads, the 26,429 nucleotide positions of each inverted repeat region were examined for differences and found to be identical. In total, 3.29 million reads (2.12%) were mapped. Median coverage was 1,999 times and the minimum coverage of any position was 600.

### Chloroplast genome of *Macadamia integrifolia *and comparative analyses

**Figure 1:**
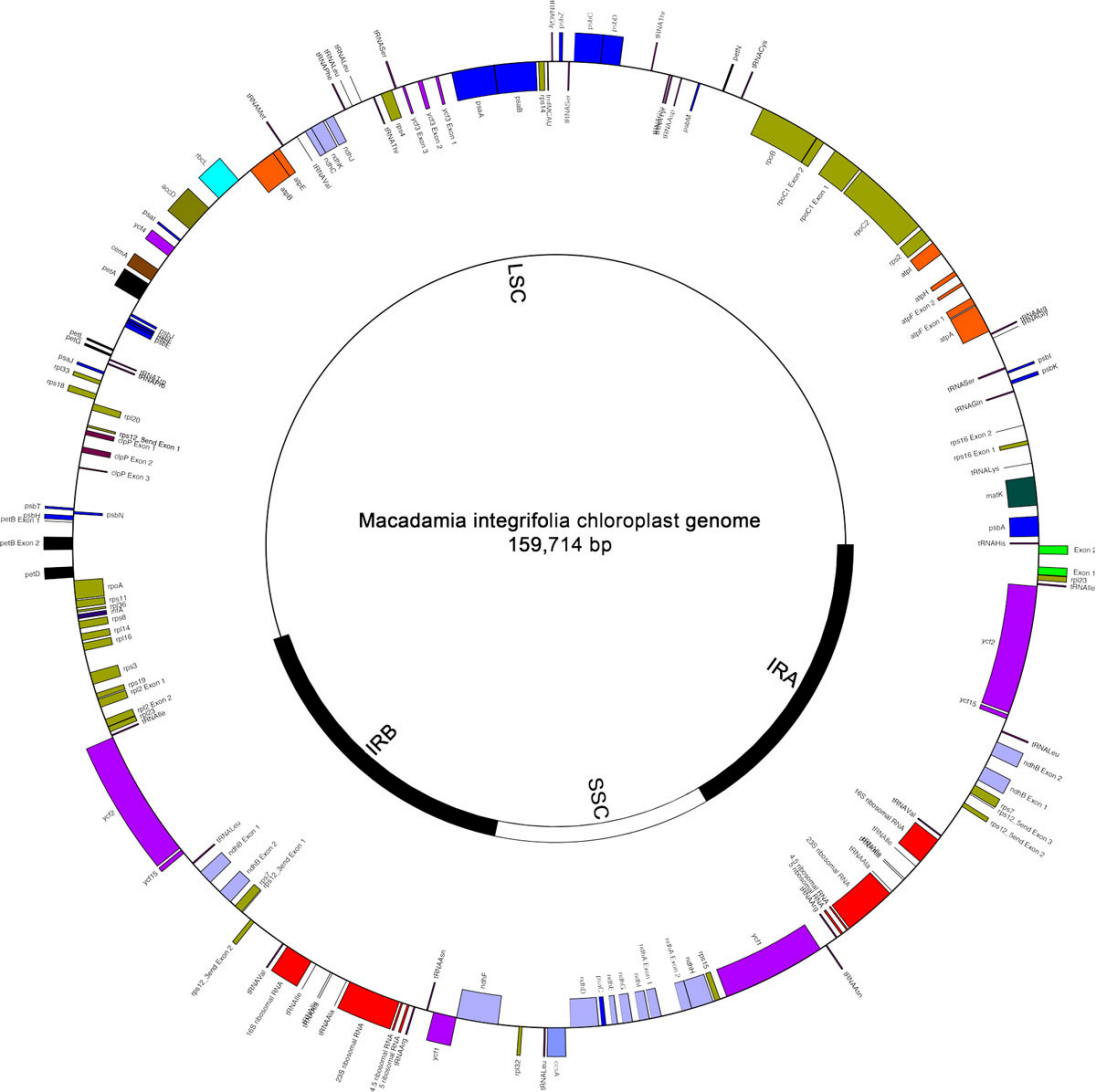
**Chloroplast genome map of *Macadamia integrifolia***. Organisation of the chloroplast genome of *Macadamia integrifolia *(Proteaceae) showing annotated genes. The inner circle shows the location of the large and small single copy (LSC and SSC) and inverted repeat regions (IR_A _and IR_B_).

**Figure 2:**
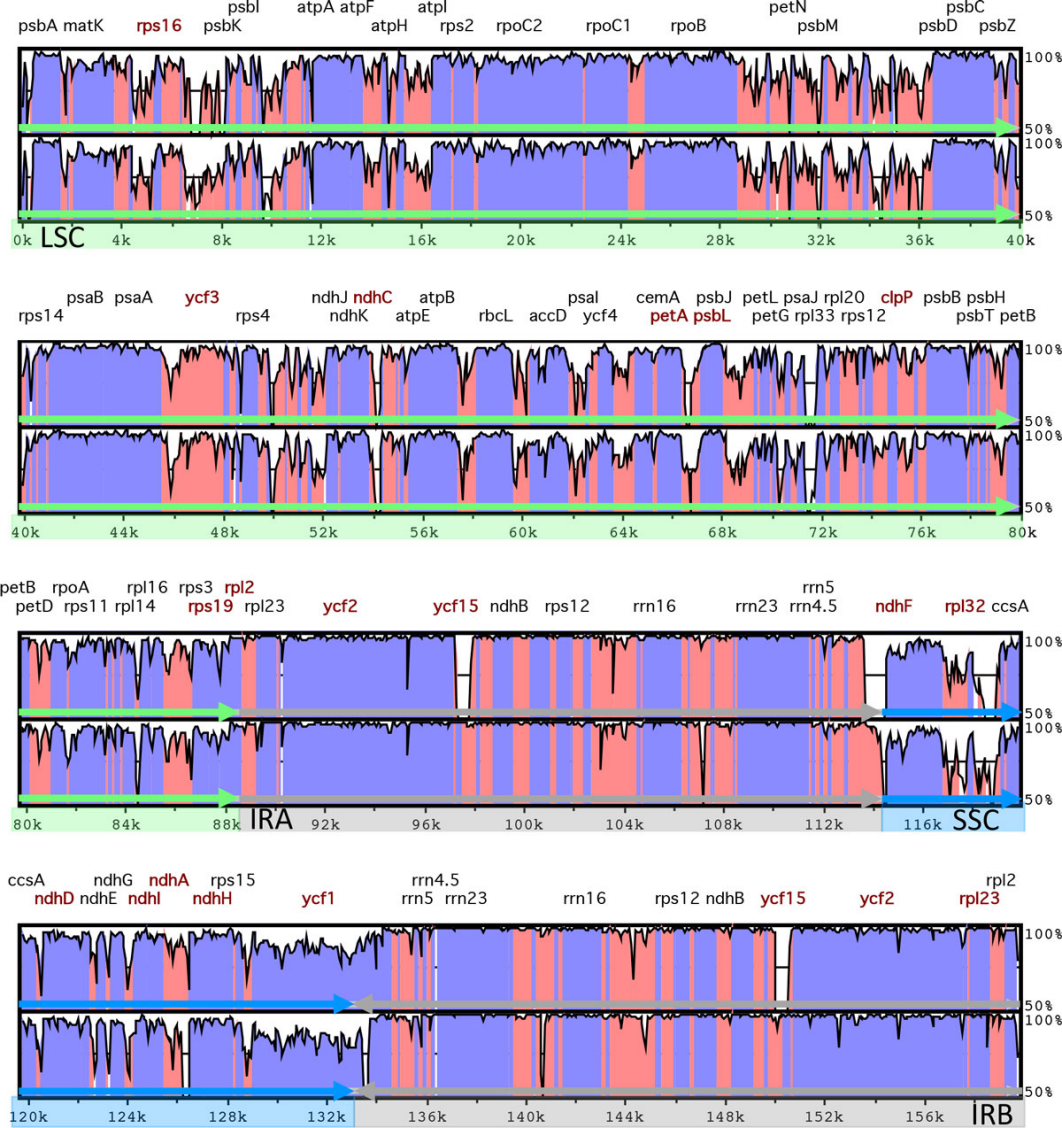
**Sequence identity plot comparing the chloroplast genome of *Macadamia *to other Proteales**. Pairwise comparisons between ***Macadamia integrifolia ***and chloroplast genomes from the Proteales genera ***Platanus ***(top) and ***Nelumbo ***(bottom) using mVISTA. The ***y***-axis represents % identity ranging from 50-100%. Coding and non-coding regions are marked in purple and pink respectively. Also shown are the genes discussed in the text (red), large single copy (LSC, green), small single copy (SSC, blue) and inverted repeat (IR, grey) regions.

The cp genome of *M. integrifolia *is 159,714 bp in length with a typical quadripartite structure [Genbank:KF862711, Figure 1]. The LSC, SSC and IR regions are 88,093, 18,813 and 26,404 bp respectively and GC content is 38.1%. Gene content and order is identical in *Macadamia, Platanus *and *Nelumbo *with each sharing 79 protein-coding, 30 tRNA and 4 rRNA genes. Size differences among Proteales cp genomes are primarily due to expansion of the IR and corresponding reductions in the LSC and SSC regions in *Macadamia *relative to *Platanus *and *Nelumbo*. Indels are located primarily in noncoding regions with the largest a 1,749 bp deletion in *Macadamia *relative to *Platanus *in the *ndhC *to *trnV-UAC *inter-genic spacer (Table 1, Figure 2). Based on internal stop codons, *ycf68 *in the *Macadamia *cp genome is a pseudogene, as in *Platanus *and *Nelumbo *and many other angiosperms. In *Macadamia ycf15 *is intact, with an amino acid sequence identical to many other angiosperms including the magnoliid *Calycanthus floridus*. In *Platanus, ycf15 *is a pseudogene [19], likely due to a 597 bp deletion in the *ycf15 *coding region relative to *Macadamia *(Figure 2). The *rps19 *gene is located at the 3'-end of the IR regions in *Macadamia *and *Nelumbo*, and spans the IR_A_-LSC and LSC-IR_B _junction in *Platanus *only. The presence of ACG start codons in *ndhD, psbL *and *rpl2 *suggests that RNA editing is required for translation of these genes in *Macadamia *and *Platanus*.

**Table 1:** Characteristics of Proteales chloroplast genomes and primary noncoding indels contributing to length differences, in base pairs

Region	*Macadamia*	*Platanus*	*Nelumbo*
Chloroplast genome	159714	161791	163307
Inverted Repeats, IR_A _and IR_B_	26404	25066	26065
*ycf2 to trnL-CAA *spacer	1014	424	1022
*trnI-GAU *intron	946	942	760
Small Single Copy, SSC	18813	19509	19330
*ndhG to ndhI *spacer	362	380	467
*ndhA *intron	2194	2187	1915
*rpl32 to trnL-UAG *spacer	1149	647	1070
Large Single Copy, LSC	88093	92150	91847
*rps16 to trnQ-UUG *spacer	1784	1299	2051
*ndhC to trnV-UAC *spacer	517	2266	2156
*ycf3 to trnS-GGA *spacer	308	889	926
*petA to psbJ *spacer	841	984	1105
*trnT-UGU to trnL-UAA *spacer	649	1419	1031
IR_B_-SSC Junction			
*trnN-GUU to ndhF *spacer	1555	746	1668
SSC-IR_A _Junction			
*ycf 1*	5538	5748	5520

### Characterisation of cpSSR loci

In total, 59 chloroplast simple sequence repeat (cpSSR) regions were identified in *Macadamia*. Of these, 57 were mononucleotide (A/T) and two were dinucleotide (AT/TA) repeats. The majority (79%), were located in noncoding sections of the LSC region. However, 14 cpSSR are located in exons including two in *ycf1 *replicated in the inverted repeat regions. No tri- or tetranucleotide repeats over 15 bp in length were found. Of particular interest for population genetics studies are regions of the *clpP *intron (660bp) and *trnK *to *rps16 *intergenic spacer (818bp) containing multiple SSRs as they are co-located in short sections amenable to PCR amplification and Sanger sequencing. The 13 cpSSRs in noncoding regions shared with *Platanus *are also of interest as they are likely to be present and may be variable in other Proteaceae species (Table 2).

**Table 2:** Distribution of *Macadamia integrifolia *chloroplast simple sequence repeat (cpSSR) regions

cpSSR	repeat motif	Length bp	start	end	Region		cpSSR	repeat motif	Length bp	start	end	Region	
1	A	15	273	288	LSC	*trnH-psbA*	32	A	10	67617	67627	LSC	*psbF *exon
2	A	10	1845	1855	LSC	*trnK-matK*	33	A	13	68887	68900	LSC	*psbE-petL*
3	A	13	4462	4475	LSC	*trnK-rps16*	34	T	12	70983	70995	LSC	*psaJ-rpl33*
4 ^b^	T	11	5269	5280	LSC	*trnK-rps16*	35	A	10	72914	72924	LSC	*rpl20-rps12*
5	T	15	6997	7012	LSC	*rps16-trnQ*	36	A	16	72943	72959	LSC	*rpl20-rps12*
6	A	10	7141	7151	LSC	*rps16-trnQ*	37 ^a,b^	T	10	74379	74389	LSC	*clpP *intron
7	A	12	9935	9947	LSC	*trnS-trnG*	38	T	10	74633	74643	LSC	*clpP *intron
8 ^b^	T	14	11345	11359	LSC	*trnG-trnR*	39	A	11	75028	75039	LSC	*clpP *intron
9	A	17	11514	11531	LSC	*trnR-atpA*	40 ^a^	A	14	81691	81705	LSC	*petD-rpoA*
10 ^a^	A	10	13186	13196	LSC	*atpA-atpF*	41 ^a^	T	13	83842	83855	LSC	*infa-rps8*
11 ^b^	A	11	14349	14360	LSC	*atpF *intron	42	T	17	84941	84958	LSC	*rpl14-rpl16*
12	T	11	15497	15508	LSC	*atpH-atpI*	43	T	12	86421	86433	LSC	*rps16-rps3*
13	T	10	17839	17849	LSC	*rps2 *exon	44	AT	16	4782	4798	LSC	*trnK-rps16 *
14	A	10	18105	18115	LSC	*rps2-rpoC2*	45	AT	16	36068	36084	LSC	*trnT-psbD*
15 ^b^	T	10	20185	20195	LSC	*rpoC2 *exon							
16 ^a,b^	T	11	20316	20327	LSC	*rpoC2 *exon	46	T	11	88100	88111	IR_B_	*rps19-rpl2*
17	T	12	22934	22946	LSC	*rpoC1 *exon	47	T	10	114378	114388	IR_B_	*ycf1 *exon
18	A	11	30325	30336	LSC	*trnC-petN*	48	T	10	114477	114487	IR_B_	*ycf1 *exon
19	T	10	32312	32322	LSC	*psbM-trnD*							
20 ^a^	T	10	34365	34375	LSC	*trnE-trnT*	49 ^b^	T	18	117406	117424	SSC	*ndhF-rpl32*
21	A	12	35944	35956	LSC	*trnT-psbD*	50	A	10	125592	125602	SSC	*ndhA *intron
22 ^a^	T	10	39082	39092	LSC	*psbC-trnS*	51	A	14	125805	125819	SSC	*ndhA *intron
23	A	16	39929	39945	LSC	*psbZ-trnG*	52	T	10	128234	128244	SSC	*ndhH-rps15*
24 ^a^	A	15	48007	48022	LSC	*ycf3 *intron	53	T	12	129915	129927	SSC	*ycf1 *exon
25	A	12	48271	48283	LSC	*ycf3-trnS*	54	A	15	130143	130158	SSC	*ycf1 *exon
26	T	14	49898	49912	LSC	*trnT-trnL*	55 ^a^	A	11	132293	132304	SSC	*ycf1 *exon
27	T	14	51057	51071	LSC	*trnL-trnF*	56	T	10	132660	132670	SSC	*ycf1 *exon
28	T	13	51972	51985	LSC	*trnF-ndhJ*							
29	T	10	52600	52610	LSC	*ndhJ-ndhK*	57	A	10	133320	133330	IR_A_	*ycf1 *exon
30 ^a,b^	T	10	55335	55345	LSC	*trnM-atpE*	58	A	10	133419	133429	IR_A_	*ycf1 *exon
31	T	14	63629	63643	LSC	*ycf4-cemA*	59	A	11	159696	159707	IR_A_	*rpl2-trnH*
cpSSR regions also present in *Platanus (a), Nelumbo (b)*													

### Phylogenetic analyses

Maximum likelihood (ML) analyses were performed on 87-taxa chloroplast gene and 160-taxa IR alignments in order to determine the position of *Macadamia *within *Angiospermae*, and the consequence of its inclusion on inferring phylogenetic relationships among basal eudicots.

#### Chloroplast Gene Phylogeny

**Figure 3:**
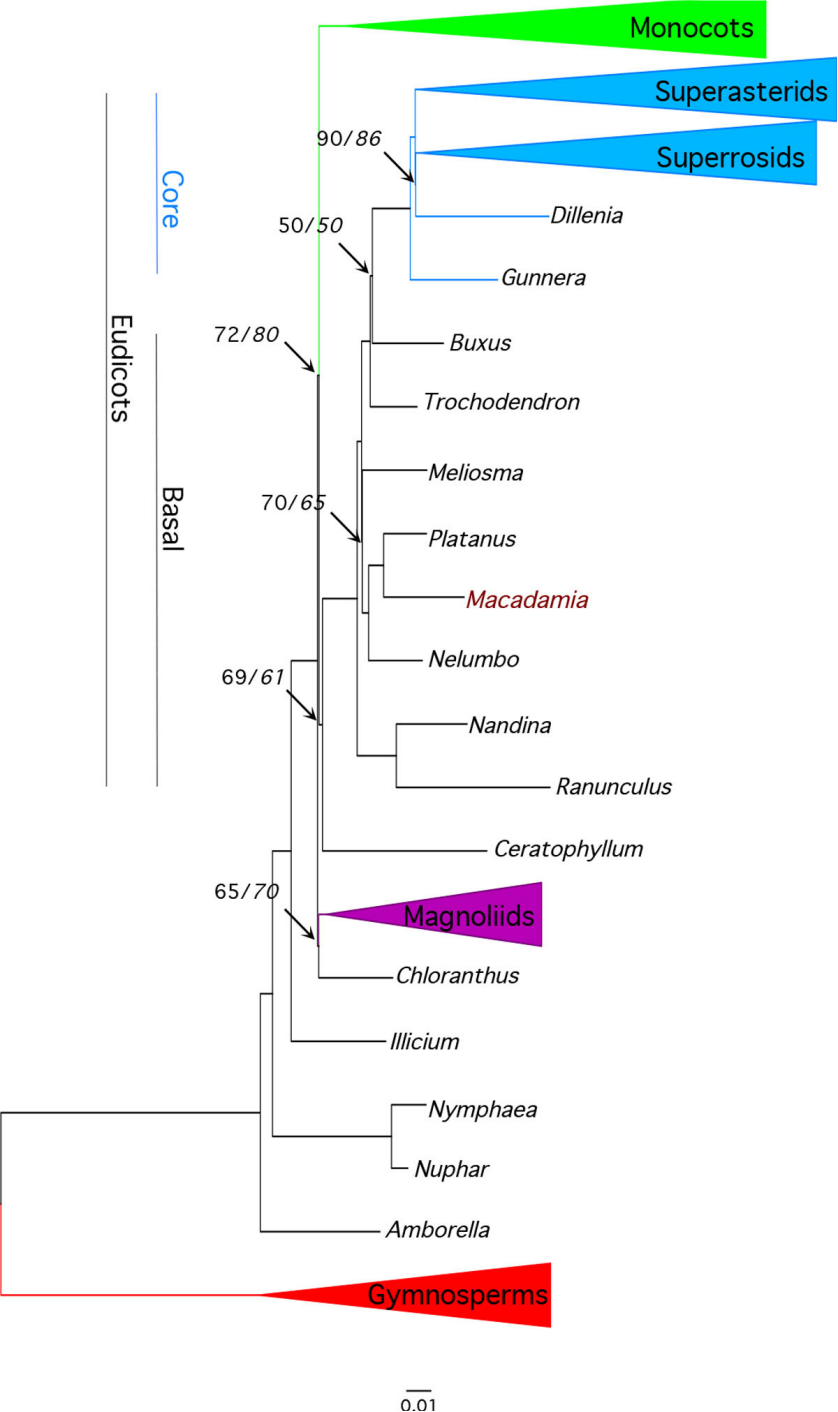
**Chloroplast gene *Angiospermae *phylogeny**. Phylogram of the best tree determined by RAxML for the 83-gene, 87-taxa, 49-partition data set. Numbers associated with branches are maximum likelihood percentage bootstrap support values for partitioned and unpartitioned (in italics) analyses. Unnumbered branches had 100% support. Collapsed monophyletic clades and number of taxa in brackets are monocots (11), superasterids (26), superosids (27), magnoliids (3) and gymnosperm outgroup (3). Scale represents substitutions per site.

The final 87-taxa and 83-gene alignment used for analyses was 66,738 bp in length. The proportion of gaps and undetermined characters was 4.04 %, and GC content was 38.4%. The optimal partitioning scheme identified under the Bayesian information criteria (BIC) using relaxed clustering analysis in PartitionFinder (lnL = -1081518.0; BIC 2170800.8) contained 49 partitions. Maximum likelihood analyses under the 49-partition and single partition (hereafter unpartitioned) strategies and the GTR+Γ model produced identical topologies. The ML 'best' tree with the highest likelihood score (lnL = -1087999.5) produced by the partitioned ML analysis (Figure 3; Fig. S2 in Additional File 2) shared the same topology as the best tree from unpartitioned analysis (lnL= -1110496.6).

#### Inverted repeat region phylogeny

**Figure 4:**
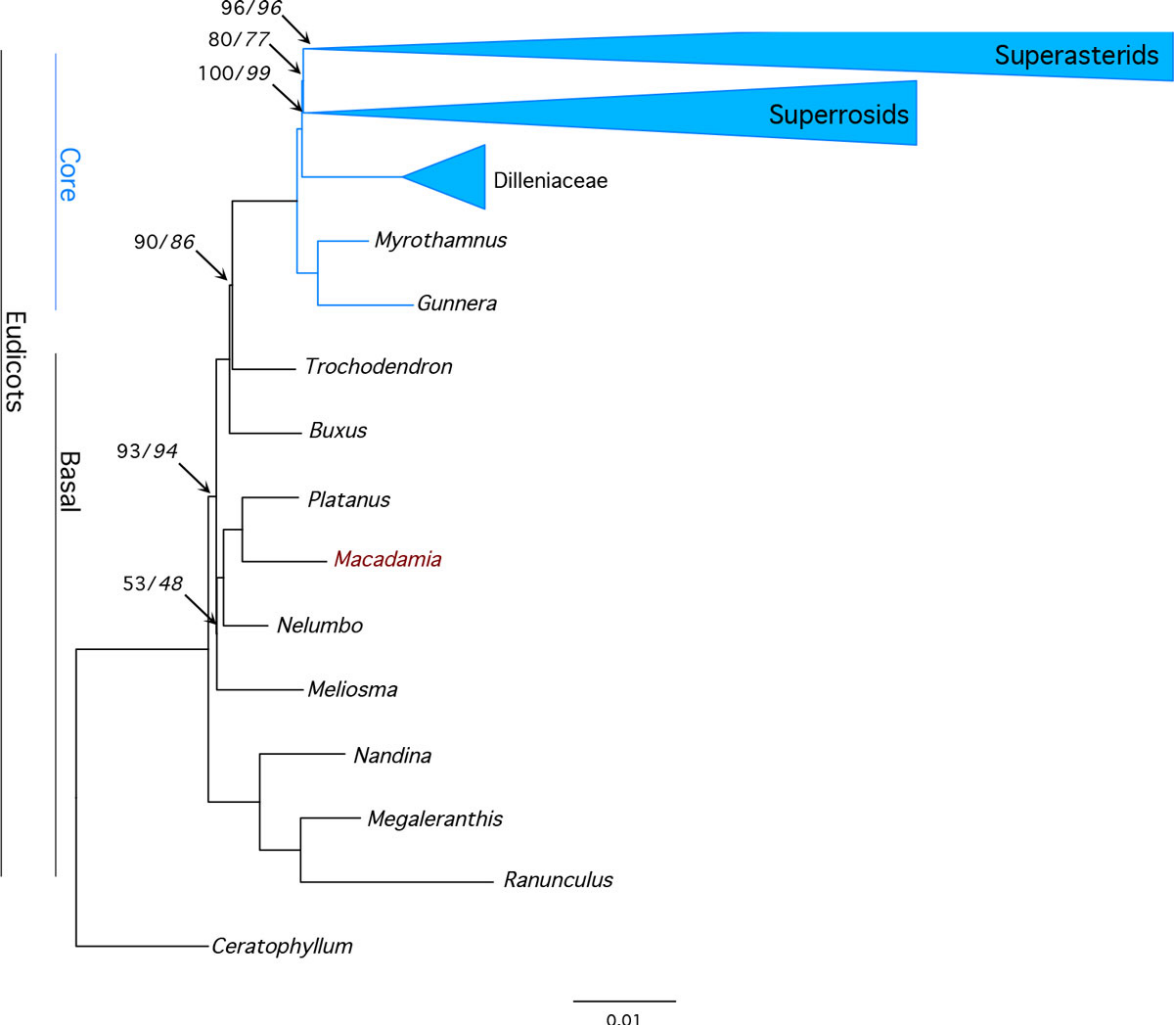
**Inverted repeat *Eudicotyledoneae *phylogeny**. Phylogram of the best tree determined by RAxML for the inverted repeat, 160-taxa, 5-partition data set. Numbers associated with branches are maximum likelihood percentage bootstrap support values for partitioned and unpartitioned (in italics) analyses. Unnumbered branches had 100% support. Collapsed monophyletic clades and number of taxa in brackets are superasterids (59), superosids (82) and Dilleniaceae (7). Scale represents substitutions per site.

The final IR alignment used for analyses was 24,693 bp in length, including 10,781 bp (43.7%) of non-coding sequence from spacers and introns. The proportion of gaps and undetermined characters was 13.2% and GC content was 42.5%. The optimal partitioning scheme in PartitionFinder (lnL = -140178.1; BIC 296837.1) contained 5 partitions. Maximum likelihood analyses under the 5-partition and unpartitioned strategies with the GTR+Γ model produced identical topologies. The ML 'best' tree (lnL = -261860.8) produced by the partitioned analysis (Figure 4; Fig. S3 in Additional File 3) shared the same topology as the best tree from unpartitioned analysis (lnL= -288975.8).

Phylogenetic analyses based on both chloroplast genes and inverted repeat regions provided maximum bootstrap (BS) support for a sister relationship between *Macadamia *and *Platanus*, and for a Proteales clade also containing *Nelumbo *(BS 100%). Sabiaceae (*Meliosma*) was sister to the Proteales in all analyses, however, the level of support for this clade was lower in the IR (BS 53%) compared to the 83-gene (BS 70%) partitioned analyses (Figure 3, Figure 4). The 83-gene and IR phylogenies were highly congruent, with the only differences among basal eudicot taxa in the position of *Buxus *and *Trochodendron*. In the 83-gene phylogeny, support for a *Buxus *versus *Trochondendron *sister relationship to the core eudicots was marginal (BS 50%), whereas *Trochodendron *was sister to the core eudicots (= *Gunneridae*) in the partitioned (BS 90%) and unpartitioned (BS 86%) IR analyses respectively. The main topological difference among core eudicots was in the position of the three major clades: superrosids, superasterids and Dilleniaceae. In cp-gene phylogenies, *Dillenia *was sister to the superrosids (BS 90%) and in IR phylogenies Dillenaceae was sister to the superosids+superasterids (BS 80%).

## Discussion

### Characteristics of the *Macadamia *cp genome and comparison to other angiosperms

The chloroplast genome of *Macadamia integrifolia *cultivar HAES 741 was sequenced at deep coverage (~2000x) and assembled *de novo *using Illumina NGS reads. Structure, gene content and order appear to be highly conserved in the basal eudicot order Proteales, and in comparision to the inferred ancestral cp genome organization of *Nicotiana tabacum *and many other angiosperms [20]. Consistently high levels of conservation within *Angiospermae *are indicative of evolutionary constraints on the cp genome of photosynthetic plants [21]. Major differences among angiosperm cp genomes are due to gene losses, inversions and expansion/contraction of inverted repeat regions. Gene loss in parasitic plants can lead to markedly reduced cp genome size. For example, the cp genome of the underground orchid *Rhizanthella gardneri *is only 59 kb [22] and there may have been a complete loss of the plastid genome in *Rafflesia lagascae *[23]. Gene loss can also be due to the transfer of cp genes to the nuclear genome [24]. Gene order is largely conserved among angiosperms including *Macadamia *and other basal eudicots, however, large inversions altering gene order have been reported in some core eudicot species [e.g. 25,26]. The main effects of expansion and contraction of the IR regions at the LSC and SSC junctions are the formation of pseudogenes, and changes in genome size and evolutionary rate [24]. The smaller cp genome of *Macadamia *compared to *Platanus *and *Nelumbo *is primarily due to relative reduction of the single copy regions, with most deletions in intergenic spacers and introns. The *Macadamia *IR at 26.4 kb is the largest yet reported in the Proteales but is considerably smaller than those of the basal eudicot *Trochondendron *(30.7 kb) and the core eudicot *Pelargonium *x *hortorum*, (76 kb) [25,27]. Proteales cp genomes also differ in the complement of pseudogenes. The intact *ycf15 *gene of *Macadamia *is, in *Platanus *a pseudogene due to a large deletion. The function and validity of *ycf15 *are uncertain, and there is no evidence of chloroplast-nuclear gene transfer in angiosperms with intact or disabled *ycf15 *genes [28]. The *rps19 *gene spans the IR_A_-LSC junction causing an pseudogene in the IR_B _of *Platanus*.

### Phylogenetic implications and the position of Proteaceae

Australia is the origin and centre of diversity of the Proteaceae, and this morphologically distinct and diverse family is distributed across remnant landmasses of the southern supercontinent Gondwana [15]. The order Proteales inclusive of Proteaceae, Platanaceae and Nelumbonaceae was established relatively recently, on the basis of molecular data, and morphological synapomorphies for the order are yet to be identified [29-31].

The 83 cp gene and IR region alignments used in this study are the largest sequence-rich datasets to include the basal eudicot family Proteaceae. Maximum likelihood phylogenies confirmed the position of *Macadamia *within the Proteales, and were congruent and largely concordant with recent phlyogenomic studies [1,3-6]. There was maximum support for a sister relationship between Proteaceae and Platanaceae, and for a Proteales clade containing these families and Nelumbonaceae. A 640-taxa angiosperm phylogeny using 17 genes from all three plant genomes included the Proteaceae taxa *Petrophile *and *Roupala *[6], and a 57-taxa, 17 kb alignment of chloroplast introns, spacers and genes included *Embothrium *and *Grevillea *[32]. Both studies confirmed inclusion of Proteaceae in the Proteales (BS 100%), in accordance with the Angiosperm Phylogeny Group III system [10]. In this study, a clade containing Sabiaceae (*Meliosma*) and Proteales was recovered from both the 83-gene and IR analyses with moderate support (Figure 3, Figure 4). These results are consistent with those from previous phylogenomic studies with support values ranging from 43-88%, although a Proteales-Sabiaceae clade was not recovered in all analyses [1-6,32].

The inclusion of *Macadamia *in taxon-rich chloroplast gene and inverted repeat alignments produced largely congruent and well-supported ML phylogenies. The main topological differences were in the positions of taxa representing lineages that are unplaced in the APG III system including *Dillenia, Trochodendron *and *Buxus *[10]. Within core eudicots, there was conflicting strong support for a sister relationship between (1) *Dillenia *and superrosids, and (2) Dillenaceae, represented by 7 genera, and superosids+superasterids, in cp-gene and IR analyses respectively. There was strong support for *Trochodendron *as sister to core eudicots in IR analyses, whilst the core eudicot sister was undetermined between *Trochodendron *and *Buxus *in cp-gene analyses (BS 50%). Interestingly, previous studies provided strong support for a Buxaceae-core eudicot clade based on data from the cp, mitochondrial and nuclear genes [6] and for an alternative *Trochodendron*-core eudicot clade using the cp IR region. Efforts to resolve relationships among unplaced angiosperm lineages, are hampered by short internal branch lengths due to rapid divergence of major lineages in the Cretaceous [7]. Full resolution of relationships among basal eudicots may require denser sampling of both taxa and genes.

### Utility of the *Macadamia *chloroplast genome

Problems in identfying a single locus DNA barcode for plants, and advances in sequencing technologies have led to suggestions that the cp genome could have utility in species identification [33,34]. Possible obstacles include the cost and complexity of assembly [35]. However, the advantages of using a NGS approach to chloroplast DNA barcoding include the potential to eliminate PCR and hence reliance on 'universal' primers. Given the widely reported transfer of chloroplast sequence to the nuclear genome [36,37] avoidance of PCR further eliminates the risk of amplifying paralogous nuclear plastid-like sequences (NUPTs). The availability of high quality cp genomes for representatives of each of the 413 recognised angiosperm families should facilitate species identification. This can be achieved through rapid identification of cp sequences by reference mapping of low coverage NGS reads at multiple locations, without the requirement for complete genome assembly. Continual improvements in sequencing technologies, including increased read lengths and decreasing cost, in addition to new methods to optimise recovery of chloroplast sequences from plant DNA [38,39] are bringing cp genome-wide barcoding closer to reality.

Whole chloroplast genome sequencing enables identification of intraspecific variation for phylogeographic studies, even in genetically depauperate species [40] and cpSSR regions have been widely used in population genetics [41,42]. The 59 *Macadamia *cpSSR identified in this study may provide markers with broad utility across Proteaceae species. The *Macadamia *cp genome is currently providing a reference sequence for inferring the domestication and evolutionary histories of *Macadamia *(unpublished results). Furthermore, it will be of benefit for taxon-rich phylogenomic studies and understanding of the evolution and adaptations underlying the remarkable diversity of this large Southern Hemisphere plant family [31].

## Conclusions

The complete chloroplast genome of *Macadamia integrifolia *was assembled *de novo *from Illumina NGS reads, and provides the first reference genome sequence for the Gondwanan plant family, Proteaceae. Despite sequencing at deep coverage (~2000x) the genome was recovered in three contigs, one of which corresponded to a collapsed copy of the inverted repeat regions. Although genome assembly from these contigs was straightforward, this provides an illustration of the problems that large repeat regions present to *de novo *genome assembly from NGS short read sequence data. Phylogenetic analyses of both 83-gene and inverted repeat region alignments confirmed the position of Proteaceae in the order Proteales, with maximum support for a sister relationship between Platanaceae (*Platanus*) and Proteaceae (*Macadamia*). The *Macadamia *chloroplast genome provides a high-quality reference for future evolutionary studies within the Proteaceae and will be of benefit for taxon-rich phylogenomic analyses aiming to resolve relationships among early-diverging angiosperms and more broadly across the plant tree of life.

## Methods

### Sample Collection

Fresh leaf material was collected from a single *Macadamia integrifolia*, cultivar 741 'Mauka' individual from the Macadamia Varietal Trial plantation M2 at Clunes, New South Wales and stored at -80ºC prior to DNA extraction. A voucher specimen was deposited in the Southern Cross University herbarium [accession PHARM-13-0813].

### DNA extraction, library preparation and Illumina sequencing

Leaf tissue was frozen in liquid nitrogen and ground using a tissue lyser (MM200, Retsch, Haan, Germany). Total genomic DNA was extracted using a DNeasy Plant Maxi kit (Qiagen Inc., Valencia, CA, USA) and quantified using a Qubit dsDNA BR assay (Life Technologies, Carlsbad, CA, USA). Genomic DNA was sheared using a Covaris S220 focused-ultrasonication device (Covaris Inc., Woburn USA) to a mean fragment size of 500 bp. A DNA library was prepared using Illumina TruSeq DNA Sample Preparation kit v2 following manufacturer's instructions (Illumina, San Diego, USA). Fragment size distribution and concentration were determined using a DNA 1000 chip on a Bioanalyser 2100 instrument (Agilent Technologies, Santa Clara, USA). Approximately 4 pmol of the library was paired-end sequenced (150 × 2 cycles) on an Illumina GA IIx instrument. Base calling was performed with Illumina Pipeline version 1.7.

### *De novo *assembly of chloroplast genome

Paired-end sequence reads were trimmed to remove low quality bases (Q<20, 0.01 probabilility error) and adapter sequences in CLC Genomics Workbench, version 4.9 (CLC Bio, Aarhus, Denmark; http://www.clcbio.com). CLC *de novo *assembler, which utilises de Bruijn graphs for the assembly, was used for the assembly with the option to map reads back to contigs and the following optimised parameters: k-mer = 35; bubble size = 50; indel cost =3; length fraction = 0.5; similarity index = 0.8. In order to identify contigs of cp origin, assembled sequences were aligned to a local database containing angiosperm complete cp genome sequences from NCBI using BLASTN [43]. Contigs with significant alignment were selected for further analysis. Alignments were visualised using mummer dotplots [44] to estimate the proportion of the genome covered and to order and connect contigs. Quality trimmed reads were mapped back to contigs using Burrows-Wheeler Aligner [45] and contigs were extended and joined using Gap5 [46]. Coverage and quality of the draft genome sequence were assessed by reference mapping of trimmed paired-end reads using CLC Genomics Workbench. To confirm accuracy, sequences spanning contig and repeat region junction regions were PCR amplified using custom primers and Sanger sequenced. The finished genome was annotated using DOGMA (Dual Organellar Genome Annotator) [47] and deposited in Genbank [Genbank:KF862711].

### Phylogenetic and Comparative Analyses

To compare structure and gene content within the order Proteales, and to identify variable regions the annotated cp genomes of *Macadamia integrifolia, Platanus occidentalis *[DQ923116] and *Nelumbo nucifera *[FJ754270] were aligned using MAFFT version 7.017 [48]. A visual representation of the alignment and regions of interspecific variation was generated in mVISTA [49]. Chloroplast SSR regions were identified using Msatcommander version 1.8.2 specifying minimum lengths of 10, 16, and 24 bp for mono-, di-, and tri- and tetranucleotides respectively [50].

To examine the position of Proteaceae within *Angiospermae*, genes were extracted from the *Macadamia *cp genome and added to the 83-gene, 86-taxa alignment of Moore et al. [4] in Geneious Pro, version 7.1.5 (Biomatters Ltd., Auckland, New Zealand). The gymnosperm outgroup taxa were *Pinus, Cycas *and *Ginkgo*. To further examine relationships among basal eudicot taxa, the slowly-evolving IR region of the *Macadamia *cp genome was aligned to a 159-taxa eudicot subset of the inverted repeat alignment of Moore et al. [5] in Geneious Pro with the outgroup taxon *Ceratophyllum*. Regions of alignment ambiguous sequence data in both matrices, and insertions present in one or few taxa were excluded. For each alignment, PartitionFinder version 1.1.1 was used to select the best-fit partitioning scheme under the Bayesian information criterion with the relaxed clustering algorithm, default 10% [51]. Data blocks analysed in the 83-gene alignment included first, second and third codon positions for 79 protein-coding genes, and 4 ribosomal RNA genes. Data blocks analysed in the IR alignment included codon positions for 7 protein-coding genes, and 4 ribosomal RNA genes, 7 transfer RNA genes, intergenic spacers and introns. Maximum likelihood analyses on partitioned and unpartitioned datasets were conducted using Randomised Accelerated Maximum Likelihood, RaxML version 7.4.2 with 100 bootstrap replicates and 10 subsequent thorough ML searches under the general time reversable (GTR) substition model and the gamma (Γ ) model of among site rate heterogeneity [52]. Bootstrap proportions were drawn on the tree with highest likelihood score from the 10 independent searches. Trees were visualised in FigTree version1.4.0.

## Competing interests

The authors declare that they have no competing interests.

## Authors' contributions

CN and GK conceived and designed the study. CN performed *de novo *assembly, genome annotation, phylogenetic and other analyses and drafted the manuscript. AB participated in bioinformatics, genome assembly and phylogenetic analyses. All authors read and approved the final manuscript.

## Supplementary Material

Additional File 1 **Figure S1: Dot plot analysis of *Macadamia *chloroplast contigs**. Dotplot showing identify of three ***Macadamia ***de novo assembled chloroplast contigs in comparison to the chloroplast genome of ***Platanus occidentalis*.** Click here for file

Additional File 2 **Figure S2: Phylogram of the best ML tree determined by RaxML (lnL = -1087999.5) for the 83-gene, 87-taxa and 49-partition data set**. Numbers associated with branches are ML percentage bootstrap support values. Click here for file

Additional File 3 **Figure S3: Phylogram of the best ML tree determined by RAxML (lnL = -261860.8) for the inverted repeat region, 160-taxa and 5-partition data set**. Numbers associated with branches are ML percentage bootstrap support values. Click here for file

## Declarations

The publication costs for this article were funded by Southern Cross University, Lismore, Australia.

This article has been published as part of *BMC Genomics *Volume 15 Supplement 9, 2014: Thirteenth International Conference on Bioinformatics (InCoB2014): Computational Biology. The full contents of the supplement are available online at http://www.biomedcentral.com/bmcgenomics/supplements/15/S9.
