# Supplementary figures and images for: Complete chloroplast genome of Macadamia integrifolia confirms the position of the Gondwanan early-diverging eudicot family Proteaceae
Source: BMC Genomics. 2014 Dec 8;15(Suppl 9):S13. doi: 10.1186/1471-2164-15-S9-S13 (PMC4290595; doi:10.1186/1471-2164-15-S9-S13)

Contigs\_vs\_P\_occidentalis

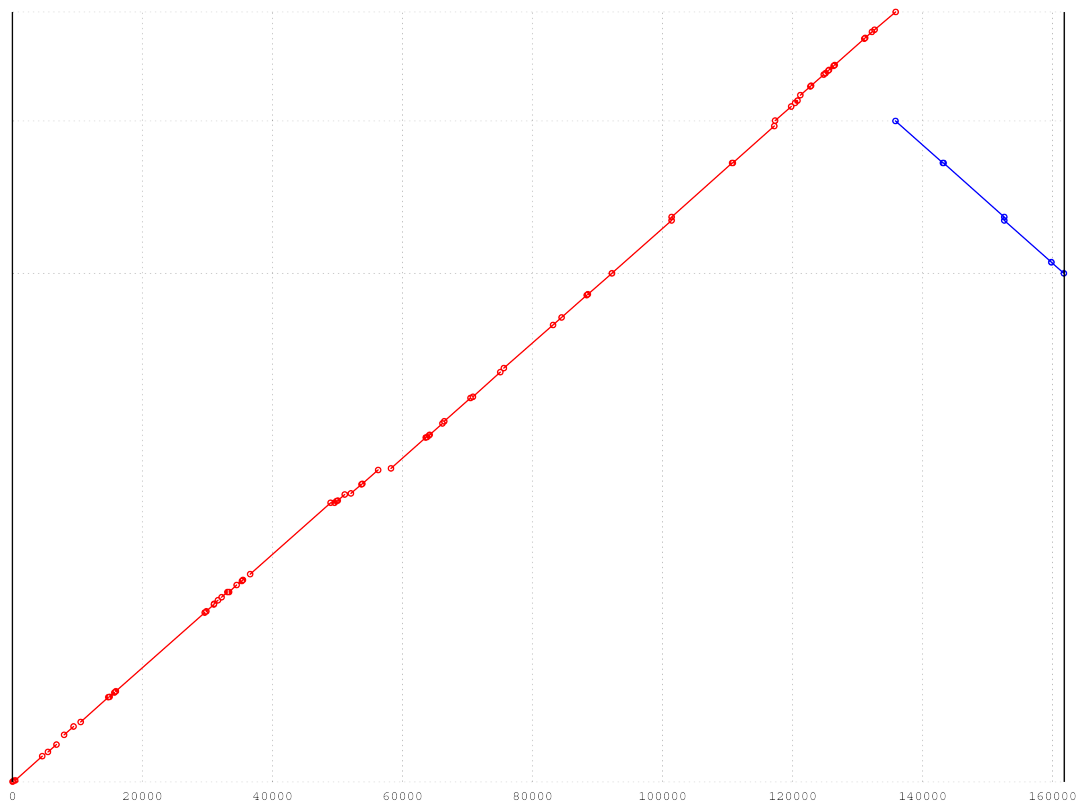

Supplement: Additional File 1 — Figure S1: Dot plot analysis of Macadamia chloroplast contigs. Dotplot showing identify of three Macadamia de novo assembled chloroplast contigs in comparison to the chloroplast genome of Platanus occidentalis. [file 1471-2164-15-S9-S13-S1.pdf]

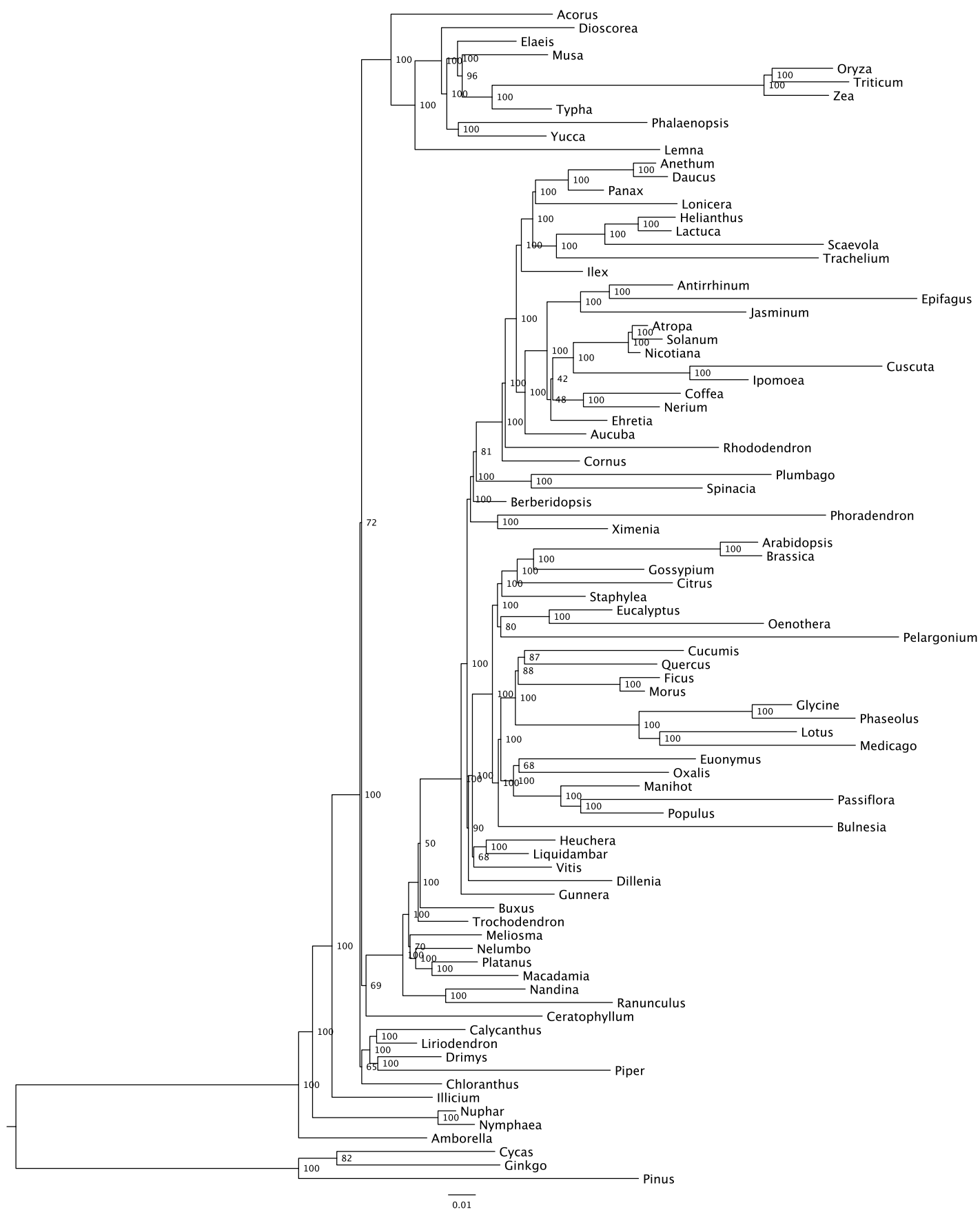

Supplement: Additional File 2 — Figure S2: Phylogram of the best ML tree determined by RaxML (lnL = -1087999.5) for the 83-gene, 87-taxa and 49-partition data set. Numbers associated with branches are ML percentage bootstrap support values. [file 1471-2164-15-S9-S13-S2.pdf]

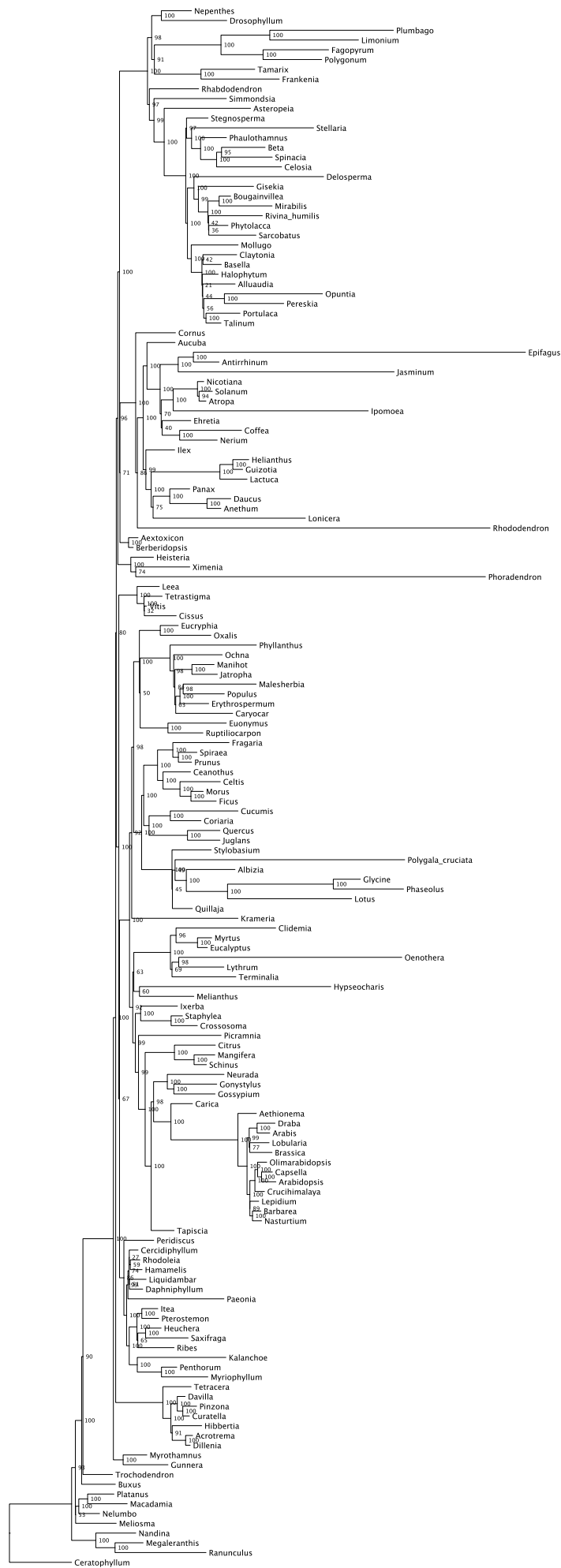

Supplement: Additional File 3 — Figure S3: Phylogram of the best ML tree determined by RAxML (lnL = -261860.8) for the inverted repeat region, 160-taxa and 5-partition data set. Numbers associated with branches are ML percentage bootstrap support values. [file 1471-2164-15-S9-S13-S3.pdf]
